# Transcriptomic Analysis Reveals a Comprehensive Calcium- and Phytohormone-Dominated Signaling Response in *Leymus chinensis* Self-Incompatibility

**DOI:** 10.3390/ijms20092356

**Published:** 2019-05-13

**Authors:** Shuangyan Chen, Junting Jia, Liqin Cheng, Pincang Zhao, Dongmei Qi, Weiguang Yang, Hui Liu, Xiaobing Dong, Xiaoxia Li, Gongshe Liu

**Affiliations:** 1 Key Laboratory of Plant Resources, Institute of Botany, the Chinese Academy of Sciences, Beijing 100093, China; sychen@ibcas.ac.cn (S.C.); lqcheng@ibcas.ac.cn (L.C.); qidm@ibcas.ac.cn (D.Q.); anda580@163.com (W.Y.); liuhuisunflower@126.com (H.L.); xiaobingdong1022@163.com (X.D.); 2 Agro-biological Gene Research Center, Guangdong Academy of Agricultural Sciences, Guangzhou 510640, China; jiajunting123@126.com; 3 College of management science and engineering, Hebei University of Economics and Business, Shijiazhuang 050061, China; zhaopincang@163.com

**Keywords:** sheepgrass (*Leymus chinensis* (Trin.), self-pollination, cross-pollination, self-incompatibility, transcriptome, molecular mechanisms

## Abstract

Sheepgrass (*Leymus chinensis* (Trin.) Tzvel.) is an economically and ecologically important forage in the grass family. Self-incompatibility (SI) limits its seed production due to the low seed-setting rate after self-pollination. However, investigations into the molecular mechanisms of sheepgrass SI are lacking. Therefore, microscopic observation of pollen germination and pollen tube growth, as well as transcriptomic analyses of pistils after self- and cross-pollination, were performed. The results indicated that pollen tube growth was rapidly inhibited from 10 to 30 min after self-pollination and subsequently stopped but preceded normally after cross-pollination. Time course comparative transcriptomics revealed different transcriptome dynamics between self- and cross-pollination. A pool of SI-related signaling genes and pathways was generated, including genes related to calcium (Ca^2+^) signaling, protein phosphorylation, plant hormone, reactive oxygen species (ROS), nitric oxide (NO), cytoskeleton, and programmed cell death (PCD). A putative SI response molecular model in sheepgrass was presented. The model shows that SI may trigger a comprehensive calcium- and phytohormone-dominated signaling cascade and activate PCD, which may explain the rapid inhibition of self-pollen tube growth as observed by cytological analyses. These results provided new insight into the molecular mechanisms of sheepgrass (grass family) SI.

## 1. Introduction

About 40% of flowering plant species and at least 100 families have self-incompatibility (SI). SI is divided into gametophytic SI (GSI) and sporophytic SI (SSI) [1]. Gramineae, including the most important cereals and forage crops, belongs to the GSI system, which exhibits at least two multiallelic and independent loci (S and *Z*) [2,3,4]. Despite numerous efforts over the last six decades, so far, none of the two loci has been clearly identified at the sequence level, and the SI response mechanism is also unclear.

Compared to the grass family, two other single multiallelic S locus GSI systems have been extensively studied at the molecular level. One is the S-RNase system, which is found in Solanaceae, Rosaceae, and Scrophulariaceae [5,6]. The stigma S-RNase and the pollen F-box protein are identified as the female and male determinants, respectively [7,8]. Each S-locus F-box (SLF) protein specifically interacts with one or more S-RNase proteins that are not their own S-haplotypes, called collaborative nonself-recognition, and then S-RNase proteins are detoxified via a ubiquitin proteasome pathway [1,9,10]. *Papaver rhoeas* is another well-characterized GSI system. The cognate interaction of the female determinant, PrsS, and the male S-determinant, PrpS, triggers Ca^2+^ signaling cascades and protein phosphorylation, subsequently altering the cytoskeleton and initiating programmed cell death (PCD) in incompatible pollen [11,12,13,14,15,16]. Brassicaceae SSI has also been intensely studied. The S-receptor kinase (SRK) and its ligand S locus cysteine-rich protein (SCR) are specifically recognized [17,18,19,20], which triggers Ca^2+^ signaling and protein phosphorylation [21,22,23]. Additional factors are also involved in SI response, including the E3 ubiquitin ligase arm repeat containing 1 (ARC1) [24], M-locus protein kinase (MLPK) [25], and thioredoxin h-like 1 (THL1) [26].

In the grass family, the location of the S and Z loci on chromosomes is known via genetic mapping despite the limited knowledge on the two loci at the molecular level. The phosphoglycoisomerase (PGI-2) isozyme and the leaf Prx-7 peroxidase gene cosegregate with the S locus in *L. perenne* and *Secale cereale* on chromosome (C) 1R [27,28]. The *Z* locus cosegregates with the beta-glucosidase and esterase 4/11 genes on C2R in *S. cereale* [29]. In addition, syntenic chromosomal locations of S and Z have been confirmed by mapping analyses in *S. cereale* [30], *P. coerulescens* [31], and *L. perenne* [32].

At the same time, a lot of work has been done to identify genes defining the S and Z loci of pollen or pistil. A putative pollen S gene (*Bm2*), encoding a functional thioredoxin protein, has been originally found to cosegregate with the S genotype in *P. coerulescens*, but subsequent mapping analysis shows that it is not located at the S locus [33,34,35]. Two F-box genes have been isolated from a partial clone carrying an anther-derived marker, which shows complete linkage to the *S* locus [36,37]. Two promising stigmatic candidate S loci genes (*Can3* and *Can94*) have also been identified in *L. perenne* [38]. Furthermore, the DUF247 gene, originally identified as a Z-linked gene, has been confirmed using a fine-mapping approach, and this gene may be involved in the male component of the S locus determination in *L. perenne* [39,40].

There are several speculations about the SI response mechanism in the grass family, but neither one has been investigated at the molecular level thus far. It has been reported that late-acting stylar inhibition of pollen tube growth in *Cynodon dactylon* is very similar to S-RNase-type GSI system [41]. Some indirect evidence shows that phosphorylation events and Ca^2+^ flux signaling may involve in pollen-pistil SI in grasses [42,43,44]. Proteolytic pathways have also been reported to be involved in rye SI [30].

SI systems arose quite late in evolution, thus explaining why closely related families do not share homologous systems [45,46]. Therefore, studies on SI should not be limited to model plants. These studies must be extended to other economically significant species in order to increase their yields or promote their cross breeding.

Sheepgrass (*Leymus chinensis* (Trin.) Tzvel.), as an economically and ecologically important perennial grass in the grass family, is widely distributed on the eastern Eurasian steppe. There is approximately 420,000 km^2^ of sheepgrass grasslands in Asia and 220,000 km^2^ in China. Sheepgrass has important value in the development of animal husbandry due to its high vegetative productivity, high protein content, and good palatability. Sheepgrass also has thick and long belowground rhizomes, and it can grow across the following diverse soil and climate conditions: extremely low temperature of −47.5 °C, drought conditions when soil moisture might be less than 6%, and NaCl and Na_2_CO_3_ concentrations of 600 and 175 mmol/L. Due to these characteristics, sheepgrass plays important roles in ecological protection, especially in soil and water conservation [47]. However, the low seed-setting rate is a prominent problem in sheepgrass for seed production over a long period of time. 

Our previous study revealed that sheepgrass is a GSI species, in which most self-pollinated pollen grains are incompatible but cross-pollinated pollen grains are compatible, and the seed-set range of sheepgrass ranges from 6.50% to 56.70% under cross-pollination and from 0.56% to 4.26% under self-pollination [48]. Subsequently, a transcriptomic analysis of mature stigmas, mature ovaries, and leaves were carried out and 1025 sheepgrass stigma-specific or preferentially expressed genes were identified in our laboratory [49]. However, the molecular mechanism of SI response in sheepgrass remains barely known. In the present study, to provide a global view of SI-related genes and uncover the potential molecular basis of SI in sheepgrass, a cellular and comparative transcriptomic analysis of self- and cross-pollinated pistils were performed. Cytological observation revealed that pollen tube growth was rapidly inhibited from 10 to 30 min after self-pollination but that pollen tubes grew normally after cross-pollination. Transcriptomic comparative analyses provided a pool of SI-related gene categories of signaling and metabolic pathways, and a putative SI response molecular model in sheepgrass (grass family) was presented. The expression dataset and bioinformatic analyses also provided the opportunity for insight into the complicated SI mechanism and its evolution in the grass family and other plant species.

## 2. Results

### 2.1. Pollen Tube Growth Patterns are Different in Self- and Cross-Pollination of Sheepgrass

**Figure 1:**
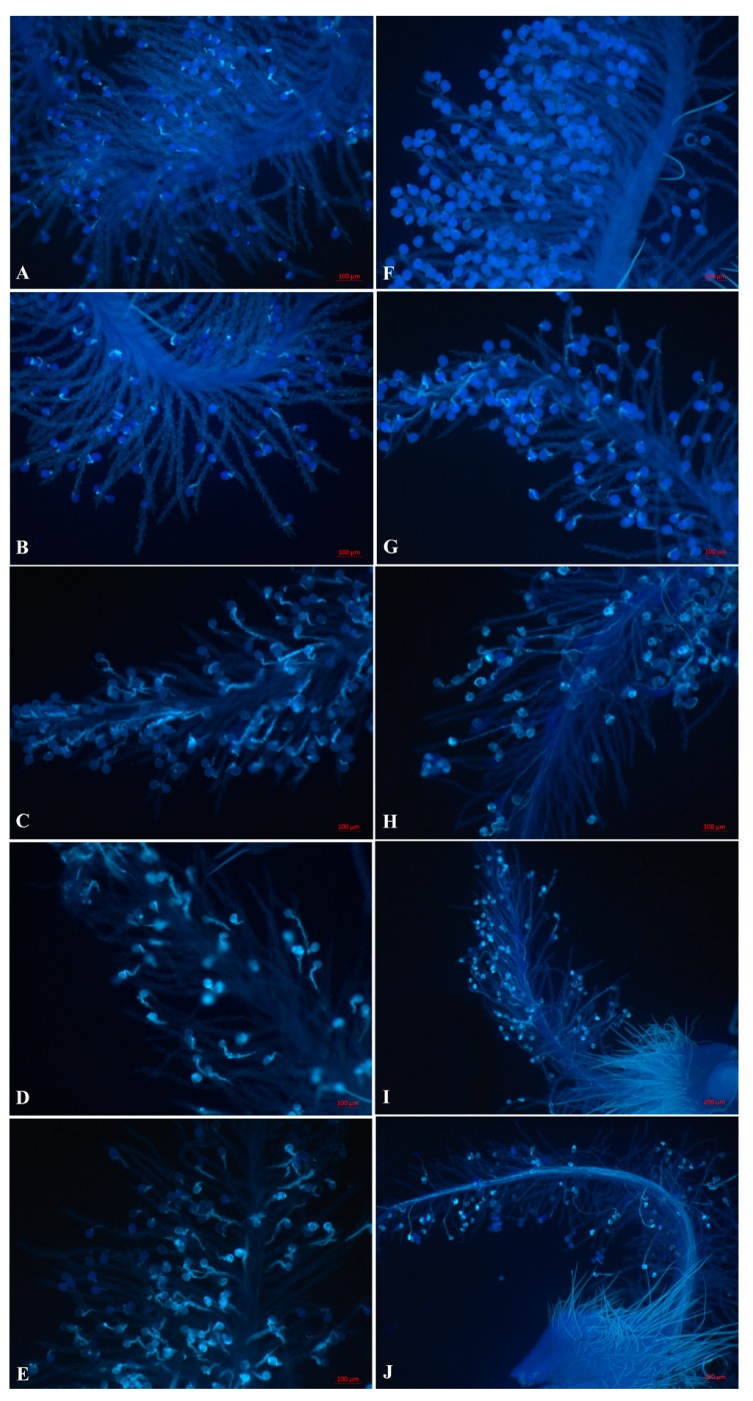
Pollen tube growth was observed under UV fluorescent microscopy. (**A**–**E**) Self-pollinated pistils at 5 min, 10 min, 30 min, 1 h, and 2 h, respectively. Pollen grains germinated normally, but the pollen tubes were soon inhibited at or near the stigma surface and may block with callose. Subsequently, pollen tubes nearly stopped growth at 30 min, and few pollen tubes reached into the style. (**F**–**J**) Cross-pollinated pistils at 5 min, 10 min, 30 min, 1 h, and 2 h, respectively. Pollen grains and pollen tubes germinated and grew normally. A majority of pollen tubes reached the style base at 1–2 h after cross-pollination. Bars: A–H, 100 μm; I–J, 200 μm.

**Figure 2:**
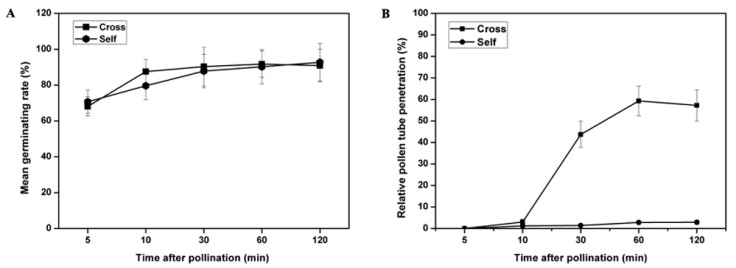
Statistical analysis of pollen germination and tube growth after self- and cross-pollination in sheepgrass. (**A**) Time course of pollen germination after pollination. No visible differences were observed in pollen grain germination for these two types of pollination. (**B**) Time course of pollen tube growth. Elongation of self-pollinated pollen tubes were inhibited from 10 to 30 min, and subsequently, the pollen tube growth was gradually stopped, while a majority of tubes reached the style base at 1–2 h after cross-pollination. Values represent the means ± SD.

Pollen grain germination and pollen tube growth were observed under UV fluorescent microscopy. Pollen grains from both self- and cross-pollination were observed on the stigma. The results showed that 70% of the pollen grains normally germinated at 5 min after self- and cross-pollination, and this number reached 88% to 90% at 30 min and then remained stable at over 90% (Figure 1). There was no significant difference in statistics between the two pollination types in pollen grain germination (Figure 2A). During pollen tube growth, self-pollinated pollen tubes were soon inhibited at or near the stigma surface and may block with callose because bright fluorescence was observed under UV light. Pollen tubes had nearly stopped their growth at 30 min after self-pollination, and few pollen tubes had reached into the style (Figure 1A–E, Figure 2B). In contrast, cross-pollinated pollen tubes grew normally. A majority of pollen tubes reached the style base at 1–2 h after cross-pollination (Figure 1F–J, Figure 2B).

### 2.2. Global Comparison of Pistil Transcriptomes during Self- and Cross-Pollination in Sheepgrass

Based on the cytological evidence of pollen tube growth, the transcriptomes of self- and cross-pollinated pistils at 5 min, 10 min, and 30 min, and of nonpollinated pistils were investigated. Each experiment was performed with two biological replicates, and the samples were sequenced using an Illumina HiSeq 4000. The sequenced reads were quality filtered (see Methods). Approximately 893 million reads passed quality control, and 134 G clean bases were obtained. The average number of quality reads per library was over 60 million (Table S1). De novo Trinity assemblies were run and yielded 399,224 transcripts with an average length of 820 bp and a N50 of 1307 bp. The longest transcript for each gene was selected as the unigene. This selection produced a total of 220,800 unigenes with an average length of 614 bp and a N50 of 901 bp (Table 1). The unigene length distribution is shown in Figure S1. All unigenes were annotated by searching against seven databases (see Methods). A total of 127,709 unigenes were annotated in at least one database, and the proportion of annotation was 57.83% (Figure S2).

**Figure 3:**
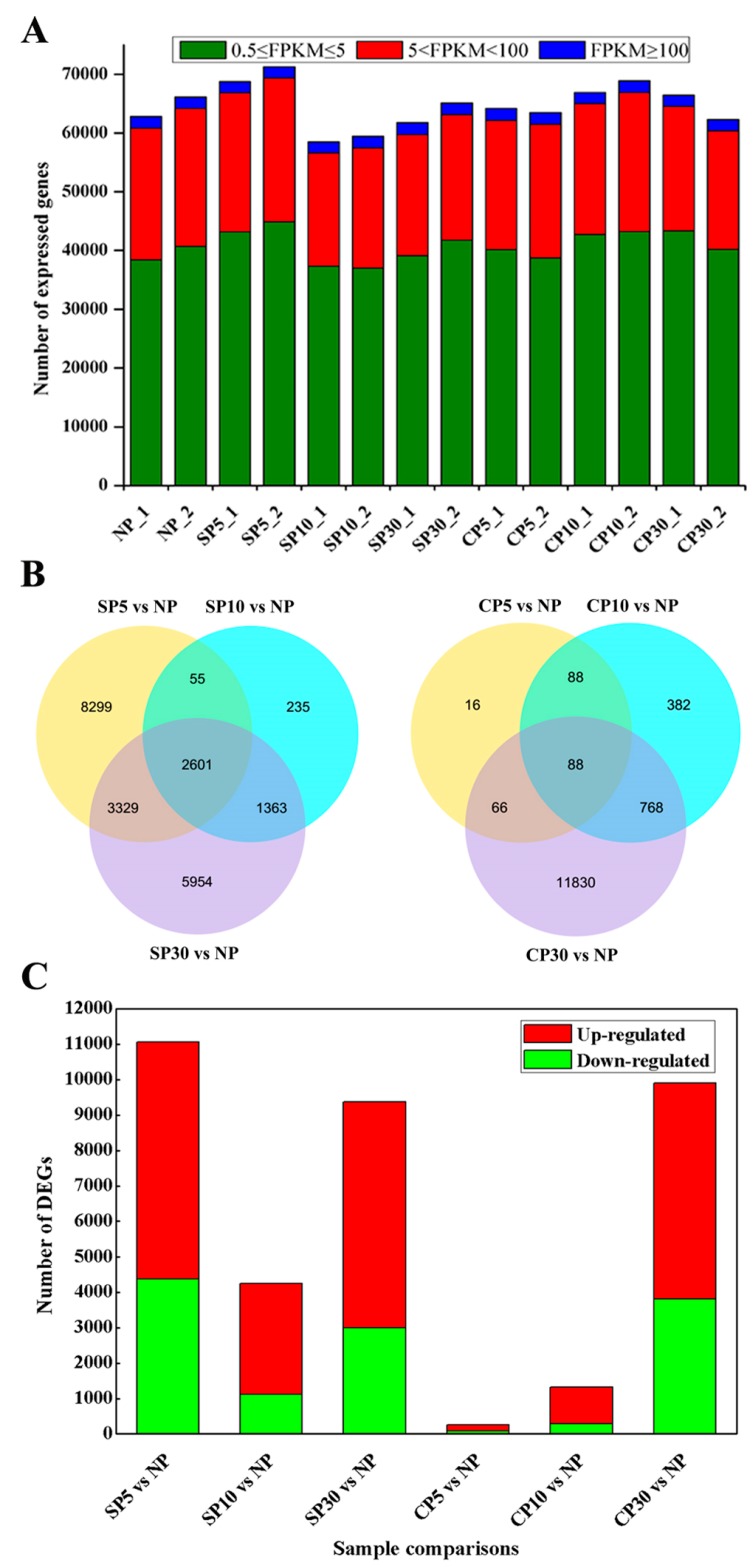
Global gene expression profiling of self- and cross-pollination in sheepgrass. (**A**) Numbers of genes expressed at different levels (based on FPKM) in the 14 samples of non-, self-, and cross-pollinated pistils. (**B**) Venn diagrams showing the numbers of differentially expressed genes (*p*-value ≤ 0.01) during each time course in self- and cross-pollinated pistils with respect to nonpollinated pistils. (**C**) Bar chart showing the number of upregulated (red) and downregulated (green) genes during each time course in self- and cross-pollinated pistils with respect to nonpollinated pistils. NP indicates nonpollinated pistils. SP5, SP10, and SP30 indicate self-pollinated pistils compared to nonpollinated pistils at 5 min, 10 min, and 30 min, respectively. Similarly, CP5, CP10, and CP30 indicate cross-pollinated pistils compared to nonpollinated pistils at 5 min, 10 min, and 30 min, respectively.

To analyze unigene expression, normalized read counts (fragments per kilobase of transcript per million mapped reads (FPKM)) for each gene were calculated, and genes with FPKM values lower than 0.5 were considered to be not expressed [50]. Based on this criterion, an average of 64,722 genes was identified as expressed in each sample. Approximately 63% of the expressed genes were in the 0.5 ≤ FPKM ≤ 5 range, and 33% of expressed genes were in the range 5 < FPKM < 100 (Figure 3A). All gene expression levels are shown in Table S2.

### 2.3. Differentially Expressed Genes are Distinct in Self- and Cross-Pollinated Pistils

Differentially expressed gene (DEG) analysis was conducted by comparing self- or cross-pollination to nonpollination. There were 11,065 (6685 upregulated and 4380 downregulated), 4254 (3144 upregulated and 1110 downregulated), and 9366 (6374 upregulated and 2992 downregulated) DEGs that responded to self-pollination at 5 min, 10 min, and 30 min, respectively. Similarly, 258 (167 upregulated and 91 downregulated), 1326 (1041 upregulated and 285 downregulated), and 9909 (6097 upregulated and 3812 downregulated) DEGs were detected in cross-pollination at 5 min, 10 min, and 30 min, respectively (Figure 3B,C). These results indicated that many DEGs were rapidly induced at 5 min after self-pollination compared to cross-pollination, in which many DEGs were induced at 30 min. A complete summary of all DEGs, both up- and downregulated, is shown in Table S3. To validate the quality of the RNA-Seq data, six DEGs were selected and performed for qRT-PCR analysis. The results showed that there was a strong correlation between RNA sequence and qRT-PCR data, which indicated the reliability of the transcriptomic data (Figure S3).

### 2.4. K-Means Clustering Analysis of DEGs

**Figure 4:**
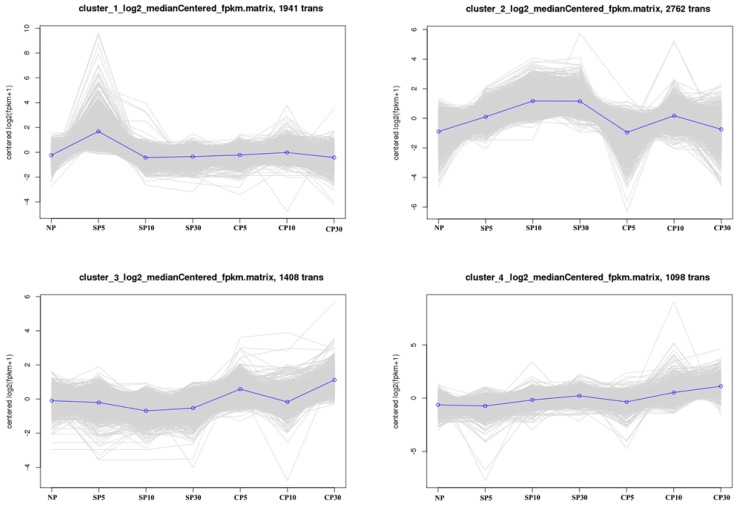
Different gene expression patterns between self- and cross-pollination as analyzed using K-means clustering. Each square represents a pattern. The horizontal axis represents non-, self-, and cross-pollination at 5 min, 10 min, and 30 min, respectively. NP, nonpollination; SP, self-pollination; and CP, cross-pollination.

Hierarchical cluster analysis of the obtained DEGs was conducted. The higher number of DEGs during self-pollination and their distinct expression patterns during self- and cross-pollination were revealed by the heatmap (Figure S4). K-means clustering analysis was performed to further identify the temporal expression patterns of genes, and this led to four clustered gene profiles for the self- and cross-pollination groups. Each profile represented a gene cluster with similar expression trends and included 1941, 2762, 1408, and 1098 unigenes. Figure 4 shows the self-pollination-specific gene clusters (cluster_1 and cluster_2) and cross-pollination-specific gene clusters (cluster_3 and cluster_4). Many SI-related genes were identified in self-pollination-specific gene clusters, including genes involved in protein phosphorylation, calcium signaling, plant hormone signaling, ROS signaling, NO signaling, and the cytoskeleton.

### 2.5. Weighted Gene Coexpression Network Analysis of DEGs

**Figure 5:**
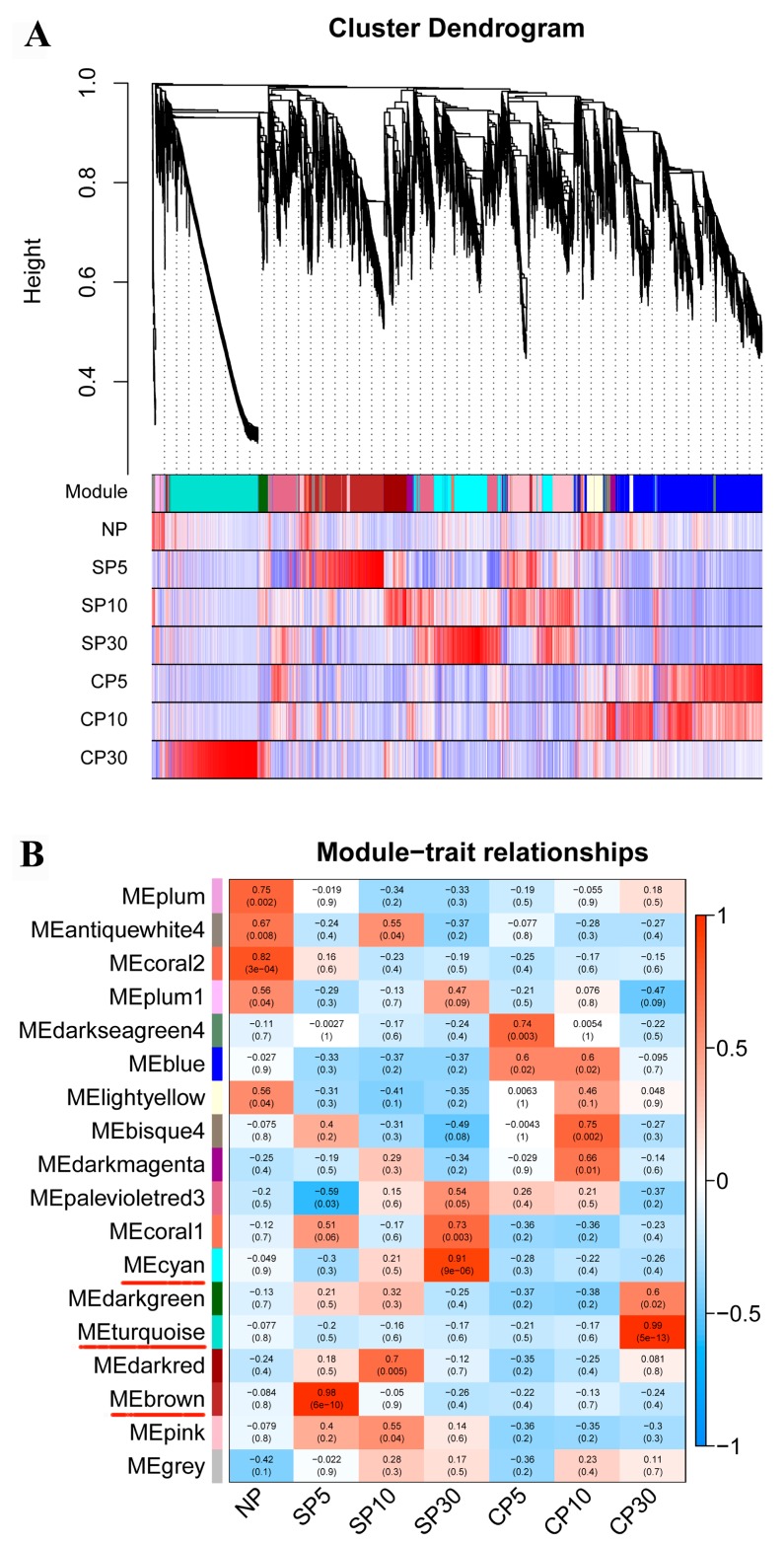
WGCNA of differentially expressed genes (DEGs) in self- and cross-pollinated pistils over time. (**A**) Hierarchical cluster tree showing coexpression modules identified using WGCNA. Each leaf in the tree represents one gene. The major tree branches constitute 18 modules labeled with different colors. (**B**) Module–sample association. Each row corresponds to a module labeled with a color as in (**A**). Each column corresponds to a specific sample. The color of each cell at the row–column intersection indicates the correlation coefficient between the module and the sample type. A high degree of correlation (>0.9) between a specific module and the sample type is indicated via red underlining of the module name.

To further identify the specific genes that are highly associated with self- and cross-pollinated pistils, a weighted gene coexpression network analysis (WGCNA) was performed. A total of 22,197 DEGs were used for the WGCNA. Coexpression networks were constructed based on pairwise correlations in gene expression across all samples. Each module was defined as a cluster of highly interconnected genes, and genes within the same cluster shared high correlation coefficients. The 18 distinct modules (labeled with different colors) were identified through this analysis, which were shown in the dendrogram in Figure 5A, in which major tree branches define the modules. The 18 modules correlated with distinct samples due to sample-specific expression profiles (Table S4). Among these, three notable coexpression modules were composed of genes that were highly expressed in self- and cross-pollinated pistils (>0.9), and these are indicated with red underlines in Figure 5B. The brown module included 1429 genes highly associated with SP5. The cyan module had 1392 identified genes and was highly associated with SP30. The turquoise module had 1766 identified genes and was highly associated with CP30. In the brown and cyan module, SI-related genes were identified, including genes involved in protein phosphorylation, calcium signaling, plant hormone signaling, and cytoskeleton.

### 2.6. Enriched Gene Ontology Terms and Kyoto Encyclopedia of Genes and Genomes Pathways of DEGs

**Figure 6:**
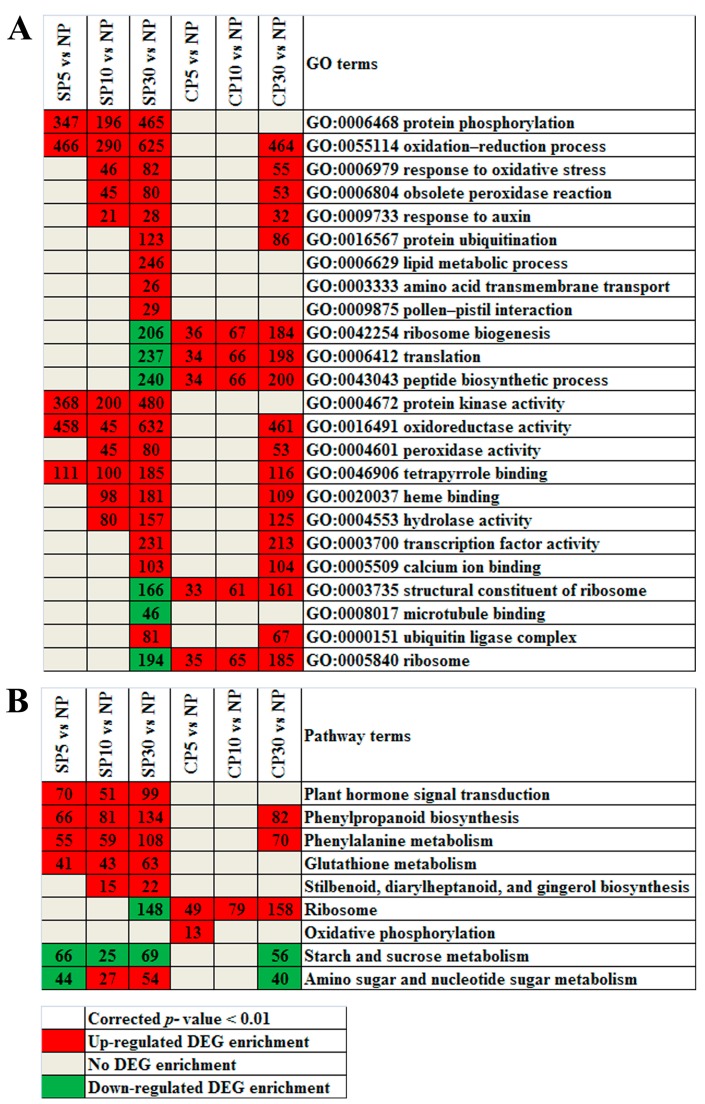
Enriched GO terms and KEGG pathways of the differentially expressed genes (DEGs). (**A**) Enriched GO terms of the DEGs. (**B**) Enriched KEGG pathways of the DEGs. GO terms and KEGG pathways with corrected *p*-value < 0.01 were considered significantly enriched. Red, upregulated DEG enrichment; green, downregulated DEG enrichment; and gray, no DEG enrichment. SP5, SP10, and SP30 indicate DEGs in self-pollinated pistils compared to nonpollinated pistils at 5 min, 10 min, and 30 min, respectively. Similarly, CP5, CP10, and CP30 indicate DEGs in cross-pollinated pistils compared to nonpollinated pistils at 5 min, 10 min, and 30 min, respectively.

To identify the biological processes, molecular functions and cellular components contributing to self- and cross-pollinated pistils, enriched Gene Ontology (GO) terms of the DEGs were determined. DEGs with a corrected *p*-value < 0.01 were defined as significantly enriched (Figure 6A). For biological processes, the specific enriched GO terms in self-pollination upregulated DEGs were protein phosphorylation, lipid metabolic process, amino acid transmembrane transport, and pollen-pistil interaction. The GO terms of ribosome biogenesis, translation, and peptide biosynthetic process were significantly enriched in cross-pollination upregulated DEGs and in self-pollination downregulated DEGs. For molecular functions, protein kinase activity was specifically enriched in self-pollination upregulated DEGs. The GO term of structural constituent of ribosome was significantly enriched in cross-pollination upregulated DEGs and in self-pollination downregulated DEGs. Similarly, for cellular components, the GO term of the ribosome was enriched in cross-pollination upregulated DEGs and in self-pollination downregulated DEGs. These results showed that the enriched GO terms of the DEGs were distinct in self-pollination and cross-pollination (Table S5).

Kyoto Encyclopedia of Genes and Genomes (KEGG) pathways with corrected *p*-value < 0.01 were defined as significantly enriched. Specifically, the enriched KEGG pathways in self-pollination upregulated DEGs were involved in plant hormone signal transduction; glutathione metabolism; and stilbenoid, diarylheptanoid, and gingerol biosynthesis. The significantly enriched KEGG pathways in cross-pollination were ribosome and oxidative phosphorylation (Figure 6B; Table S6), indicating that the enriched KEGG pathways of the DEGs were also distinct in self-pollination and cross-pollination. 

Subsequently, to obtain SI response gene categories, the results of the K-means clustering analysis, WGCNA, GO enrichment analysis, and KEGG enrichment analysis were combined with an artificial screening process. This process generated a pool of SI-related genes.

### 2.7. A Pool of SI-Related Gene Categories

#### 2.7.1. SI Triggers Protein Phosphorylation-Related Gene Expression in Sheepgrass

**Figure 7:**
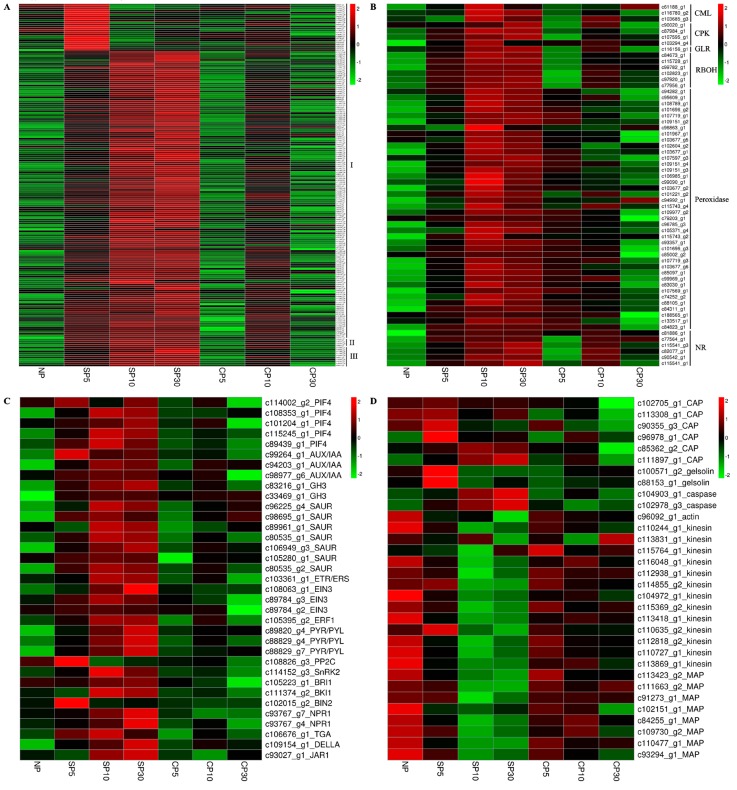
Expression profiles of selected SI response gene categories. (**A**) Protein phosphorylation (I) and putative pollen (II)–stigma (III) recognition-related genes. (**B**) Calcium (Ca^2+^), reactive oxygen species (ROS), and nitric oxide (NO) signaling-related genes. (**C**) Plant hormone signaling-related genes. (**D**) Cytoskeleton-related genes. NP, nonpollination; SP, self-pollination; and CP, cross-pollination.

Many protein phosphorylation-related genes were identified in the present study, and various types of protein kinase genes were activated. These genes included cysteine-rich receptor-like protein kinase, serine/threonine protein kinase, leucine-rich repeat receptor protein kinase, wall-associated receptor kinase, and CBL-interacting protein kinase genes, which indicated that phosphorylation events may be involved in the sheepgrass SI response (Figure 7A). 

Cysteine-rich peptides (CRPs) are one of the main classes of signaling peptides in plants and are overrepresented in SI, pollen germination, and growth. The Brassicaceae SCR protein has been identified as the male S-determinant. Three genes encoding CRP secretory proteins were detected as specifically expressed in sheepgrass self-pollination (Figure 7A). Several genes were annotated as G-type lectin S-receptor-like serine/threonine protein kinases and preferentially expressed in sheepgrass self-pollination (Figure 7A). The Pfam annotation showed that these genes contained S locus glycoprotein domains, and their GO terms have been described for pollen recognition. These results indicated that G-type lectin S-receptor-like serine/threonine protein kinases and CRP secretory proteins may be candidate stigma and pollen recognition proteins in sheepgrass. The complete list of genes is shown in Table S7.

#### 2.7.2. SI Triggers Calcium, ROS, and NO Signaling-Related Gene Expression in Sheepgrass

Calcium (Ca^2+^), as an important second messenger, is well known for its importance in SI response and the regulation of pollen tube growth. Several Ca^2+^ signaling-related genes, including a glutamate receptor-like channel (GLR) gene, three calcium-binding protein (CML) genes, and four calcium-dependent protein kinase (CPK) genes, had higher expression levels in sheepgrass self-pollination than in cross-pollination (Figure 7B). Thus, these genes may mediate sheepgrass SI response and inhibit pollen tube growth. 

ROS and nitric oxide (NO) are involved in PCD and are triggered by the SI response. Peroxisomes and respiratory burst oxidase homologs (RBOHs) are the major sources of ROS production in plant cells. In the present study, more than 30 peroxidases and 6 RBOH-related genes were detected as preferentially expressed in sheepgrass self-pollination (Figure 7B). Nitrate reductase (NR) plays a central role in plant biology. It regulates not only the levels of the NO signaling molecule, but also the amounts of nitrite that can be used for nitrogen assimilation. Six NR genes were identified with high expression levels in sheepgrass self-pollination (Figure 7B), and their potential signaling role deserves further exploration. Table S8 shows the list of genes.

#### 2.7.3. SI Triggers Plant Hormone Signaling-Related Gene Expression in Sheepgrass

Several kinds of genes related to plant hormone signal transduction were detected in the sheepgrass SI response (Figure 7C). For auxin signaling, three major classes of auxin-responsive genes and one type of auxin regulatory gene were significantly upregulated in sheepgrass self-pollination. These groups included auxin/indole-3-acetic acid (Aux/IAA), Gretchen Hagen 3 (GH3), small auxin-up RNA (SAUR), and phytochrome-interacting factor (PIF) genes. Genes involved in ethylene signal transduction, including one ethylene receptor (ETR and ERS), one ethylene-responsive transcription factor 1 (ERF1), and three ethylene-insensitive protein 3 (EIN3) genes, were also detected in the sheepgrass SI response. Several abscisic acid signaling genes, such as abscisic acid receptors (PYR/PYL), protein phosphatase 2C (PP2C), and serine/threonine protein kinase SRK2 (SnRK2), were differentially expressed in sheepgrass self-pollination compared to cross-pollination. These results indicated that plant hormones may act in the SI response. The complete list of genes is shown in Table S9.

#### 2.7.4. SI Triggers Cytoskeleton-Related Gene Expression in Sheepgrass

The plant cytoskeleton comprises actin microfilaments and tubulin microtubules. Several actin-binding proteins (ABPs) and microtubule-associated proteins (MAPs) were detected in the present study (Figure 7D). Two Ca^2+^-dependent ABPs (gelsolins) and six cyclase-associated protein (CAP) genes, which are involved in F-actin depolymerization and the formation of punctate F-actin foci, were preferentially expressed in sheepgrass self-pollination. MAPs control microtubule dynamic instability and/or interfilament cross-talk. Several MAP genes, including a kinesin family member, were downregulated in sheepgrass self-pollination. Caspase-3, one of the key mammalian executioner caspases, has a tetrapeptide recognition motif of DEVD, so is known as a DEVDase. DEVDase/caspase-like activity has been shown to be involved in SI-mediated pollen tube inhibition and early PCD events [14]. Two caspase genes were also detected in the self-pollination response. These results suggested that the sheepgrass SI response may involve changes in actin microfilaments, tubulin microtubules, and PCD, which halt the growth of pollen tubes. The complete list of genes is shown in Table S10.

### 2.8. Putative SI Response Molecular Model in Sheepgrass

**Figure 8:**
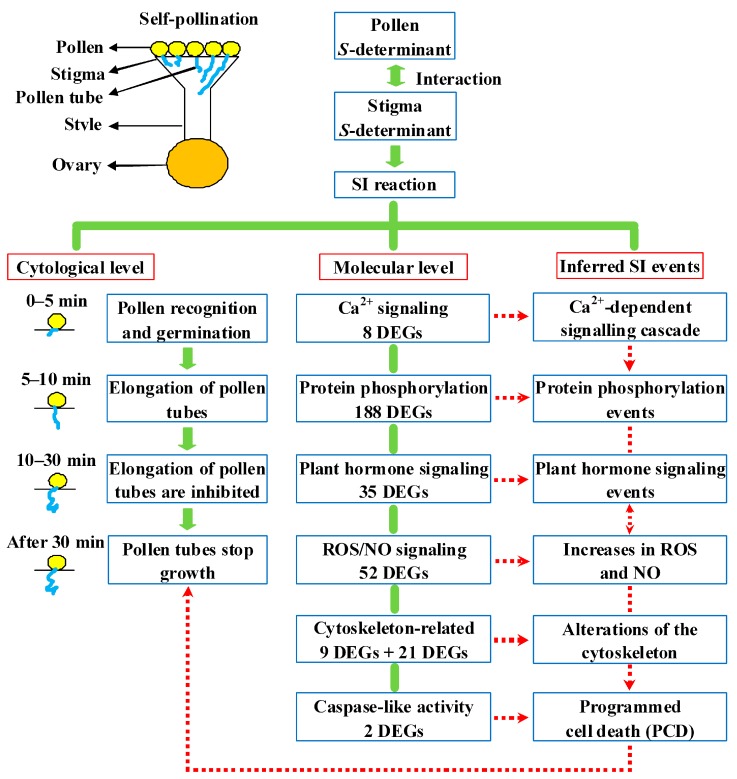
A putative molecular model of the self-incompatibility (SI) response in sheepgrass. Interaction of a stigma S-determinant with its cognate pollen *S*-determinant rapidly triggered the SI signaling network. Solid segments and arrows show SI-induced reactions at cytological and molecular levels. Dotted segments and arrows show possible SI triggered events. Cytological observation revealed that the self-pollinated pollens can normally germinate at 5 min. However, the elongation of pollen tubes was inhibited from 10 to 30 min, and subsequently, the pollen tube growth stopped. Many SI-related signaling genes and pathways were induced, including genes related to calcium (Ca^2+^) signaling, protein phosphorylation, plant hormone, reactive oxygen species (ROS), nitric oxide (NO), actin depolymerization, microtubule depolymerization, and programmed cell death (PCD). Thus, SI may trigger a comprehensive calcium- and phytohormone-dominated signaling cascade and activate PCD, which may explain the rapid inhibition of self-pollen tube growth as observed by cytological analyses.

Based on the rapid inhibition of pollen tube growth at the cytological level and the transcriptomic analysis results at the molecular level, a putative SI response molecular model in sheepgrass was generated (Figure 8). This model suggests that the SI response is initiated by a stigma *S*-determinant (receptor) and pollen *S*-determinant (ligand) interaction similar to that found in Papaver and Brassica SI. This interaction activates a series of SI signaling and metabolic genes and pathways involved in Ca^2+^ signaling, protein phosphorylation, ROS signaling, NO signaling, plant hormone signaling, cytoskeleton-related activity, and caspase-like activity. Thus, a series of SI events may be triggered. A rapid Ca^2+^-dependent signaling cascade event may trigger a rapid influx of Ca^2+^ and therefore protein phosphorylation, transient increases in ROS and NO, and plant hormone signaling. Subsequently, cytoskeleton changes occur, including actin and microtubule depolymerization. The ROS and NO signals together with caspase-like activities may activate PCD, which may explain the rapid inhibition of self-pollen tube growth as observed using cytological analyses.

## 3. Discussion

Sheepgrass is a self-incompatible species [48]. To date, however, studies of SI in forage grass species of economic significance, such as sheepgrass, have been limited due to the following factors: long generation times, polyploidy, large genomes, and few genomic information of these species; the absence of the usable genetic tools available as in model plants; and the few naturally occurring or induced mutants. Because closely related families do not share homologous SI systems [45,46], studies of SI must be extended to other species of economic significance and not limited to model plants. Sheepgrass is an economically and ecologically important perennial grass species. Because our previous study revealed that sheepgrass is a GSI species [48], the study of the SI response mechanism in this species is of great significance not only for insight into the complicated SI mechanisms in plants but to promote cross breeding. Although our previous transcriptomic analysis of sheepgrass stigmas provided several stigma candidate genes [49], there has been no more evidence that reveals the molecular basis of the SI mechanism. To this end, the present work provided a whole-transcriptome RNA-Seq analysis of gene expression in self- and cross-pollinated pistils to reveal SI response genes and potential mechanisms in sheepgrass.

### 3.1. Inhibition of Incompatible Pollen Tube Growth Is Rapid in Sheepgrass

In Gramineae, self-incompatibility reacts quickly. Within 5–10 min after pollen-stigma contact, intine-held antigens are released by the self-pollinated pollen grains of Phalaris tuberose on the stigma surface, and pollen tube growth is subsenquently controlled [51]. In the present study, the pollen tube growth was rapidly inhibited in self-pollinated stigma within 10–30 min after self-pollination (Figure 1 and Figure 2B). The sheepgrass, like P. rhoeas, has a dry stigmatic surface, while the Solanaceae has a wet, lipid-rich exudate stigmatic surface. Self-incompatible pollen tube growth was rapidly inhibited in a few minutes in sheepgrass and P. rhoeas, but relatively slow in the Solanaceae [52]. Whether there is a causal relationship between dry/wet stigma surface and inhibition of pollen tube growth needs further study.

### 3.2. Protein Phosphorylation Events Involved in the Sheepgrass SI Response

Phosphorylation events play a key role in the signaling cascades. The phosphorylation of SRK activates the degradation of proteasomal protein in Brassica SI [20], while phosphorylation of the p26 pyrophosphatases and p56 mitogen-activated protein kinase triggers the SI response in Papaver [53,54]. Application of different protein kinase inhibitors lead to an inhibition of the SI response in grasses, in that the evidence supports the involvement of phosphorylation events [42]. In the present study, the GO term of protein phosphorylation was significantly enriched in pistils of self-pollination but not of cross-pollination (Figure 6A). Furthermore, many protein phosphorylation-related genes were detected, and their expression levels were significantly upregulated in self-pollination compared to cross-pollination (Figure 7A), which inferred that SI in sheepgrass may trigger protein phosphorylation events. Among these genes, six genes encoding G-type lectin S-receptor-like serine/threonine protein kinases are intriguing because their GO biological process is listed as recognition of pollen and they possess S locus glycoprotein domains and kinase activity. These characteristics imply that G-type lectin S-receptor-like serine/threonine protein kinase genes might be candidate genes in pollen–stigma recognition, suggesting further study. Protein kinase genes with similar characteristics involved in pollen–stigma interactions have been reported in other species. The recognition specificity is achieved by the stigmatic SRK in the Brassicaceae SI system. SRK encodes a membrane-spanning serine/threonine kinase and determines the S-haplotype specificity of the stigma. In addition, the extracellular domain of SRK has high homology with the S locus glycoprotein (SLG) gene [55]. Another study has shown that two G-type lectin S-receptor-like kinases are near to the *Z* locus on perennial ryegrass linkage group 2 using GenomeZipper and that one kinase colocalizes with the locus [56]. Secreted CRPs play important roles in plant SI responses [57,58]. The pollen S-determinant of SCR/SP11 in the Brassicaceae and the stigma S-determinant of PrsS in *P. rhoeas* have been shown to encode CRPs [11,59]. Similarly, the present study identified four genes encoding CRPs. The expression of these genes was specifically triggered in self-pollinated pistils but was almost absent in nonpollinated and cross-pollinated pistils (Figure 7A), suggesting that they may be induced by self-pollination and represent sheepgrass candidate pollen determinants worthy of further study. 

### 3.3. Sheepgrass SI is Involved in Calcium, ROS, and NO Signaling

Ca^2+^, as an important second messenger, plays crusial roles on the regulation of pollen tube growth. In Papaver, the SI response is initiated by Ca^2+^ influx and an almost instantaneous increase in cytosolic free Ca^2+^ ([Ca^2+^]cyt) in incompatible pollen, which forms a “wave” in the shank of the pollen tube and triggers a Ca^2+^-dependent signaling cascade [60]. Several Ca^2+^ signaling-related genes, including a GLR, four CPK and three calmodulins (CMLs), were detected in the present study as preferentially expressed in self-pollinated pistils (Figure 7B). Similar genes have been reported in the sheepgrass stigma transcriptome [49]. Previous reports have also showed that these Ca^2+^ signaling-related genes are associated with SI response. In the Brassicaceae, Ca^2+^ influx into stigma papilla cells mediated by the GLR gene triggers SI signaling and leads to self-pollen rejection [23]. CPK is a major calcium sensor involved in pollen tube growth regulation. In *L. perenne*, one CPK gene (*Can94*) has been identified via a suppression subtractive hybridization experiment, and it is a stigmatic candidate gene for *S* loci [38]. Overexpression of *PiCPK1* and *PiCPK2* from Petunia inflate disrupts growth polarity or inhibits growth of the pollen tube via excessive Ca^2+^ accumulation in the tip [61]. Another study has shown that a calcium-dependent protein kinase (CPK32) interacts with CNGC18 to combine Ca^2+^ signaling with pollen tube tip growth [62]. CML, as another Ca^2+^ signal transduction sensor, regulates SI pollen tube growth by increasing Ca^2+^ influx and ROS concentration in *Pyrus pyrifolia* [63]. Application of Ca^2+^ channel blockers in *S. cereale* (rye) alters SI, indirectly indicating the involvement of Ca^2+^-mediated signaling in SI [42]. The present study observed that the growth of pollen tubes was inhibited in self-pollinated pistils but that pollen tubes grew normally in cross-pollinated pistils (Figure 1 and Figure 2B). The results of previous studies and the present study suggested that the sheepgrass SI response leads to Ca^2+^ influx in the cytosol of the pollen tube, triggering a Ca^2+^-dependent signaling cascade that affects the growth polarity or inhibits the extension of pollen tubes during self-pollination. 

SI responses involving ROS and NO production have been reported in the following three different SI systems: the Papaver GSI, the S-RNase-based Rosaceae GSI, and the SI response in olive [64,65,66]. Several important genes contribute to ROS or NO production. The activities of RBOH and peroxidase may participate in the production of ROS [65]. NR has been proposed as an important enzymatic source of NO and may play a central role in plant biology by controlling NO levels [67]. In the present study, genes involved in both ROS and NO production including a number of genes encoding RBOH, peroxidase, and NR were preferentially expressed in self-pollinated pistils (Figure 7B), which indicated that ROS and NO may be involved in the sheepgrass SI response. 

### 3.4. Roles of Plant Hormones in the Sheepgrass SI Response

In the present work, three major classes of auxin response genes and one type of auxin regulatory gene, including the Aux/IAA, GH3, SAUR, and PIF gene families, were significantly upregulated in sheepgrass self-pollination compared to cross-pollination (Figure 7C), indicating that auxin and its signaling transduction genes may be involved in the sheepgrass SI response. Several previous studies have also mentioned auxin changes during self- or cross-pollination. In Theobroma cacao, auxin increases significantly after compatible pollination [68], while another study has shown that auxin content improves after incompatible pollination [69]. In the Brassicaceae, overexpression of auxin response factor 3 (ARF3) enhances the SI response and simultaneously downregulates auxin responses in stigma epidermal cells [70]. Ethylene and abscisic acid (ABA) have been reported to exhibit a strong increase after incompatible pollination in *T. cacao* [68]. In the present work, several ethylene and ABA signaling-related genes were also detected, and their expression levels were higher in self-pollinated pistils than in cross-pollinated pistils. These genes included ethylene receptor (ETR, ERS), ethylene-responsive transcription factor 1 (ERF1), ethylene-insensitive protein 3 (EIN3), ABA receptors (PYR/PYL), protein phosphatase 2C (PP2C), and the serine/threonine protein kinase SRK2 (SnRK2) (Figure 6C). Similarly, plant hormone signaling transduction genes have been detected in tomatoe SI response [71]. Cross-talk among ROS, NO, and hormones has been well characterized in response to environmental factors, including second messengers, such as kinases or Ca^2+^ [72,73], but that has scarcely been insight into SI. The present data suggested that cross-talk among ROS, NO, and hormones may exist in the sheepgrass SI response. 

### 3.5. Sheepgrass SI May Trigger Alterations to the Cytoskeleton and PCD

The plant cytoskeleton, including actin microfilaments and tubulin microtubules, are highly dynamic by interacting with various ABPs and MAPs [74]. The actin cytoskeleton, as a possible early target of SI signals, involves Ca^2+^ signaling and inhibits pollen tube growth. In the present study, two gelsolins and six CAP genes were upregulated in self-pollinated pistils (Figure 7D). Gelsolin is a Ca^2+^-dependent ABP. A previous study has shown that a putative gelsolin (PrABP80) is implicated in SI-induced actin depolymerization [75]. The other two ABPs, actin depolymerizing factor (ADF/cofilin) and CAP, rapidly colocalize with actin foci and make actin aggregate to punctate F-actin foci [76]. Therefore, sheepgrass SI may involve Ca^2+^-dependent signaling and F-actin depolymerization to stop pollen tube growth. 

Microtubules are dynamic filaments, and the assembly and disassembly of which are under precise control by various MAPs. In the present study, many MAP genes, including kinesin family members, were detected, and their expression levels were downregulated in the sheepgrass SI response (Figure 7D). In Arabidopsis thaliana, armadillo repeat domain-containing kinesins (ARK1) may induce microtubule depolymerization because ARK1 mutants display disrupted microtubule organization [77]. The FOP-related protein of 20 kDa (FOR20), another MAP, functions to promote microtubule depolymerization and cell migration [78]. Therefore, the present results indicate that sheepgrass SI may also involve microtubule depolymerization.

Several SI-induced events are involved in PCD. ROS and NO have been considered as crucial molecules in triggering different types of PCD in plants [76]. The alterations of actin and microtubules are related to mediating PCD [79,80,81]. Caspase inhibitor and substrate experiments prove that DEVDase/caspase-like activity is involved in SI-mediated pollen tube inhibition, DNA fragmentation, and early PCD events [82,83]. In the present work, genes involved in ROS, NO, actin, and microtubule alterations were triggered by the sheepgrass SI response. Furthermore, changes in caspase gene expression were detected in sheepgrass self-pollinated pistils (Figure 7D). These data indicated that sheepgrass may use PCD to terminate the incompatible pollen tube growth.

## 4. Materials and Methods

### 4.1. Plant Materials

Two sheepgrass germplasms, Zhongke No. 1 and K1, were used. Zhongke No. 1 is an improved variety certificated by the National Grass Seed Certification Committee in China in May 2014. K1 is a new, improved strain. The original source of the K1 wild plant material was collected in Meir’s village (Meir’s District, Qigihar City, Heilongjiang Province, China) and approved by the National Animal Husbandry Service, Ministry of Agriculture and Rural Affairs of the People’s Republic of China. The self-seed-setting rate of each genotype was almost zero, but the cross-seed-setting rate of these two genotypes was higher than 75%, indicating that the *S* and *Z* allele compositions in Zhongke No. 1 and K1 may be complementary. Therefore, Zhongke No. 1 was self-pollinated, while Zhongke No. 1 and K1 were cross-pollinated. For the in vitro pollination method, mature pistils were placed on petri dishes filled with medium (2.5% agar, 25% sucrose, and 25 ppm boric acid) to support the pistils, which made the feathery stigmas easy accept incoming pollen. Mature pollen without anthers was collected in clear bags and shaken onto the surface of the plates [38]. For the in vivo pollination method, the pistils were harvested at intervals of 5 min, 10 min, and 30 min after self- and cross-pollination. Nonpollinated pistils of Zhongke No. 1 were collected as control samples. Each sample had two biological replicates. After harvesting, a portion of the materials was fixed in formalin:acetic acid:alcohol (FAA) at 5/5/90 (*v/v*). The remaining materials were stored at −80 °C for RNA extraction.

### 4.2. Observation of Pollen Tube Growth

The pistils were taken out from FAA and soaked with 3 M NaOH for 3 h. The pistils were then stained with 1% aniline blue for 3 h. Finally, the pollen tubes were observed using a Zeiss Axio Imager A1 fluorescence microscope (Carl Zeiss AG, Oberkochen, Germany).

### 4.3. RNA Extraction, Library Construction, and Sequencing

Total RNA was extracted using a TRIzol reagent (Sigma Aldrich, St. Louis, MO, USA) according to the manufacturer’s instructions. RNA integrity was measured using an Agilent 2100 Bioanalyzer (Agilent Technologies, Santa Clara, CA, USA). cDNA libraries were generated using a TruSeq Stranded mRNA sample preparation kit (Illumina) using an Illumina HiSeq 4000 system, and 150 bp paired-end reads were generated for each library. All library construction and Illumina sequencing were performed at Novogene Bioinformatics Technology Co. Ltd. (Beijing, China). 

### 4.4. De Novo Assembly and Functional Annotation

The raw data were filtered by removing reads containing adapters, reads containing poly-N, and low-quality reads, and then clean reads were obtained. Sequencing quality was evaluated by calculating the values of Q20, Q30, GC content, and sequence duplication level of the clean data. Transcriptome assembly was performed using the Trinity program [84]. The longest transcript of each gene was identified as the unigene. Seven databases were used for functional annotations of the unigenes, including NCBI nonredundant protein (Nr), NCBI nonredundant nucleotide (Nt), Protein family (Pfam), Clusters of Orthologous Groups of proteins (KOG), Swiss-Prot protein (Swiss-Prot), KEGG Ortholog database (KO), and GO database.

### 4.5. Differential Expression, Cluster Analysis, and GO Enrichment Analysis

The reads per sample were counted and mapped back to the assembled transcriptome using Trinity with RSEM software [85]. The read number of each gene was transformed into FPKM (fragments per kilobase of transcript sequence per million base pairs sequenced) for analysis of the gene expression level [86]. To identify DEGs, the DESeq program was used for differential expression analysis [87]. Unigenes meeting the criteria of an FDR adjusted *p*-value < 0.05 and |log_2_Ratio| > 1 were extracted [88]. GO and KEGG enrichment analyses were performed using GOseq and KOBAS 2.0 (http://kobas.cbi.pku.edu.cn/), respectively [89,90]. Clustering was performed using the K-means clustering algorithm [91]. WGCNA was performed using an R package [92].

### 4.6. Quantitative Real-Time PCR Analysis

Quantitative real-time polymerase chain reaction (qRT-PCR) analysis was performed to verify the transcriptome data. One microgram of total RNA was transcribed to cDNA following the PrimeScript™ RT Reagent Kit protocol (Takara, Dalian, China). qRT-PCR was conducted using the SYBR® PrimeScriptTM PCR Kit (Takara) on a Roche LightCycler 480 (Roche, Rotkreuz, Switzerland) according to the manufacturer’s instructions. The sheepgrass actin gene was used as an internal control. The relative expression level of each gene was assessed in three technical replications and determined using the 2^−ΔΔCT^ method. The primers used for qRT-PCR are listed in Table S11.

## 5. Conclusions

The present study investigated the growth and inhibition of pollen tubes using microscopic observation and the expression patterns of genes using RNA-Seq after self- and cross-pollinations. The present study provided a pool of SI-related gene categories and presented a putative SI response molecular model in sheepgrass (grass family). The model proposes that SI triggers a rapid Ca^2+^-dependent signaling cascade, which triggers the changes of gene expression level involved in protein phosphorylation as well as ROS, NO, and plant hormone signaling. Subsequently, the gene changes for cytoskeleton together with caspase-like activity may activate PCD, and these results may explain the rapid inhibition of self-pollen tube growth as observed by cytological analyses. The proposed SI response mechanisms in sheepgrass revealed using transcriptomic and cytological analysis are similar to those of Papaver SI. Our results provided data for further insight into the molecular mechanisms of sheepgrass (grass family) SI. 

## Figures and Tables

**Table 1:** De novo transcriptome assembly statistics for sheepgrass.

Metric	Value
General assembly statistics	
Total transcripts	399,224
Smallest transcript length	201
Largest transcript length	13,765
Transcript N50	1307
Transcript N90	331
Median transcript length	507
Average transcript length	820
Total transcript assembled bases	327,421,926
Statistics based on the longest transcript per gene	
Total unigenes	220,800
Unigene N50	901
Unigene N90	255
Median unigene length	352
Average unigene length	614
Total unigene assembled bases	135,549,030

## Supplementary Materials

Supplementary materials can be found at https://www.mdpi.com/1422-0067/20/9/2356/s1. Table S1. Transcriptome sequencing output statistics. Table S2. All gene expression levels. Table S3. List of differentially expressed genes (DEGs). Table S4. Gene list of 18 modules in self- and cross-pollinated pistils. Table S5. Enriched GO categories of the DEGs. Table S6. KEGG enrichment results of the DEGs. Table S7. DEGs involved in protein phosphorylation triggered by SI. Table S8. DEGs involved in calcium, ROS, and NO signaling triggered by SI. Table S9. DEGs involved in plant hormone signaling triggered by SI. Table S10. DEGs involved in cytoskeleton triggered by SI. Table S11. Primers used in qRT-PCR analysis. Figure S1. The number and length distribution of assembled unigenes. Figure S2. Unigene annotation using seven databases. Figure S3. qRT-PCR validation of expression profiles of DEGs. Figure S4. Hierarchical cluster heatmap comparison of the differentially expressed genes. Distinct expression patterns were revealed for non-, self-, and cross-pollination at 5 min, 10 min, and 30 min, respectively. NP, nonpollination; SP, self-pollination; and CP, cross-pollination.

## Abbreviations

SI	Self-incompatibility
SSI	Sporophytic self-incompatibility
GSI	Gametophytic self-incompatibility
PM	Plasma membrane
sPPase	Soluble inorganic pyrophosphatase
ARC1	Arm repeat containing 1
MLPK	M-locus protein kinase
THL1	Thioredoxin h-like1
ROS	Reactive oxygen species
NO	Nitric oxide
PCD	Programmed cell death
SLF	S locus F-box
FPKM	Fragments per kilobase of transcript per million mapped reads
DEGs	Differentially expressed genes
WGCNA	Weighted gene coexpression network analysis
GO	Gene Ontology
KEGG	Kyoto Encyclopedia of Genes and Genomes
CRPs	Cysteine-rich peptides
SCR	S locus cysteine-rich
SRK	S-receptor kinase
GLR	Glutamate receptor-like channel
CML	Calcium-binding protein
CPK	Calcium-dependent protein kinase
RBOH	Respiratory burst oxidase homolog
NR	Nitrate reductase
Aux/IAA	Auxin/indole-3-acetic acid
GH3	Gretchen Hagen 3
SAUR	Small auxin-up RNA
PIFs	Phytochrome-interacting factors
ETR, ERS	Ethylene receptor
ERF1	Ethylene-responsive transcription factor 1
EIN3	Ethylene-insensitive protein 3
PYR/PYL	Abscisic acid receptors
PP2C	Protein phosphatase 2C
SnRK2	Serine/threonine protein kinase SRK2
ABPs	Actin-binding proteins
MAPs	Microtubule-associated proteins
CAP	Cyclase-associated protein
Ca^2+^	Calcium
NAA	1-Naphthaleneacetic acid
ARF3	Auxin response factor 3
